# Developing a media literacy-based e-cigarette education program via medical record systems

**DOI:** 10.18332/tpc/201477

**Published:** 2025-04-04

**Authors:** Hongying Daisy Dai, Ellen Kerns, Hana Niebur, Ashley Deschamp, Rachel Johnson, Kaeli Samson, James Buckley, Summer Woolsey

**Affiliations:** 1 College of Public Health, University of Nebraska Medical Center, Omaha, United States; 2 Population Dynamics and Health Lab, College of Public Health, University of Nebraska Medical Center, Omaha, United States; 3 Department of Pediatrics, University of Nebraska Medical Center, Omaha, United States

**Keywords:** adolescent, media literacy, e-cigarette, anti-marketing education, interactive e-learning, electronic health record

## Abstract

**INTRODUCTION:**

This is a prospective, interventional pilot study that seeks to evaluate the impact of MediaSense, a media-literacy-based vaping prevention program, in adolescents including an oversample of those with asthma.

**METHODS:**

During July and December 2022, participants in Nebraska were recruited via electronic health record (EHR)-based messaging, and MediaSense was self-administrated by interactive e-learning with REDCap surveys before and after the intervention. Regression analysis evaluated changes in vaping media literacy, vaping expectancy, and harm perception pre- and post-intervention. Factor analysis was conducted on 22 items on usability, to determine which latent factors were most related to interactive e-learning modules.

**RESULTS:**

Adolescents aged 12–17 years participated in the MediaSense intervention (n=67; 59.7% with asthma). The pre- and post-intervention surveys showed a 148% increase in vaping media literacy (ranging 0–6; 2.9 vs 4.5, p<0.0001). Vaping expectancy (ranging1–5) decreased from 3.6 to 1.2 (p<0.0001), and the perception of vaping as harmful rose from 40.3% to 86.0% (p<0.0001). Participants rated the intervention highly on usability, technical assistance, design, content clarity, navigation, flow, multimedia, interactivity, and learning outcomes. Two distinct factors were identified in the factor analysis: motivating and engaging content (Factor 1) and user-friendly module design (Factor 2). Participants with higher usability ratings of the e-modules (Factor 1: B=0.6; 95% CI: 0.3–0.9, p=0.0004; Factor 2: B=0.7; 95% CI: 0.4–1.0, p=0.0001), and those with asthma (vs no asthma: B=0.5; 95% CI: 0.1–0.9, p=0.01) had significantly higher vaping refusal and media literacy.

**CONCLUSIONS:**

The MediaSense program demonstrated acceptability and feasibility in recruiting and preventing adolescent vaping through EHR and digital interventions. Media literacy helps adolescents to critically evaluate vaping-related marketing messages, resist persuasive marketing, and make informed decisions.

## INTRODUCTION

The e-cigarette (EC) industry has heavily marketed its products through social media, leading to a surge in teen vaping^1^. Vaping poses significant public health risks, including exposure to nicotine and toxic substances, increased respiratory and cardiovascular issues, potential nicotine addiction, and harm to brain development^2^. Tobacco marketing exposure is linked to tobacco initiation, frequency, and relapse. Recently, EC manufacturers have increased advertising, targeting youth through retail stores, the internet, streaming services, and print media^3^. EC is often marketed as a healthier alternative to cigarettes, with appealing designs and a variety of flavors that are attractive to youth^4^. Improving media literacy – understanding and evaluating vaping-related media – can help adolescents avoid EC use and recognize the risks hidden in vaping advertisements^5,6^.

Adolescents with chronic medical conditions are particularly vulnerable to e-cigarettes as alternative tobacco products^7^. Asthma, a prevalent chronic inflammatory airway disease, is characterized by variable airflow limitations due to airway narrowing and wall thickening, leading to symptoms such as shortness of breath, chest tightness, and cough. Asthma affects approximately 8.4% of US adolescents and young adults aged 11–21 years (3.8 million), highlighting its widespread impact^8^. Moreover, vaping is prevalent among asthma adolescents with an estimated 7.5% reporting current use. Meta-analyses have also established a significant association between youth vaping and an increased risk of asthma, emphasizing the compounded risks for this vulnerable population^9^. Therefore, it is imperative to develop targeted vaping prevention programs specifically designed for adolescents, including those with asthma conditions, to mitigate these risks and promote better respiratory health.

While most existing school-based programs focus on educating youth about the risks of vaping and encouraging them to avoid initiation or stop vaping, few focus on the influence of media on youth vaping behaviors^10^. Furthermore, despite schools showing strong support for addressing youth vaping, few have implemented comprehensive e-cigarette prevention and cessation programs. The main challenges for current school-based vaping prevention efforts include limited time, insufficient knowledge, lack of coordinated efforts, and poor teacher commitment^11,12^.

The development of interactive e-learning lessons that can be self-administered by youth and delivered remotely could significantly increase the reach and adoption of vaping prevention as part of digital interventions. Electronic health records (EHR) also offer a unique opportunity to reach adolescent e-cigarette users. In 2023, 95% of children aged <18 years had a visit with a doctor or other healthcare professional in the past year^13^. The EHR contains a wealth of clinical data that could be used to streamline the identification of eligible adolescents. The utilization of EHR in the recruitment of vaping interventions can not only be a cost-effective way with a high reach to young vapers, but also be scalable to other pediatrics primary care clinics.

Media Education for Sensible Evaluation and Nurturing Substance-free Experiences (MediaSense) is an anti-vaping media literacy program designed to prevent vaping among adolescents and young adults. Following CDC and FDA guidelines, MediaSense was developed using the Theory of Reasoned Action (TRA), which links intention and behavior influenced by marketing and media^14,15^ (see the Supplementary file for the process and theoretical framework of MediaSense development). Our prior study (Phase I) has shown promising results of MediaSense on improving media literacy knowledge and harm perception and reducing youth susceptibility to vaping^16^. This study (Phase II) aims to assess the feasibility of delivering MediaSense through EHR recruitment and digital intervention and evaluate the preliminary intervention effects.

## METHODS

### Step 1: Development of interactive e-learning modules

After developing the curriculum topics, the research team worked with the e-learning center at the University of Nebraska Medical Center (UNMC) to create educational materials and further graphically develop e-learning modules corresponding to nine topic areas. The process begins with a needs assessment and planning phase where specific learning objectives are defined, and the characteristics of the target audience, such as adolescents, are analyzed. Content development follows, including storyboarding to outline the flow of content and the creation of instructional materials like text, multimedia elements, and interactive features such as quizzes and simulations.

The research team assembled a 12-member intervention expert panel (IEP) comprising experts in media literacy, youth vaping epidemiology, nicotine addiction, tobacco prevention and cessation, anti-marketing communications, and tobacco control policy. The IEP was responsible for iteratively reviewing and refining the e-learning modules, focusing on defining vaping prevention topics and developing anti-marketing strategies. Modules cover essential topics such as understanding e-cigarette products, nicotine addiction, health impacts, myths versus facts in marketing, and the role of advertising. Each module consists of approximately 20 minutes of core content, including didactic slides and videos, with quizzes provided to assess understanding.

### Step 2: Reach to youth through EHR

Children’s Nebraska (CN) hospital served as the primary recruitment site. CN is the only freestanding pediatric hospital in the state of Nebraska, and it serves patients referred from throughout the regions of five states (NE, IA, SD, KS, and CO) for medical, behavioral, and social services. In 2022, these clinics provided primary care to nearly 100000 children through more than 288000 patient visits.

From July to December 2022, participants in Nebraska were recruited through the MyChart patient portal, which is embedded in the EHR system, allowing patients to see their medical records and send and receive secure messages (SM) for study enrollment. A study invitation was sent out using MyChart SM to all teenagers with an active MyChart account who met the basic criteria. The MyChart account may be controlled by the teen or their parent, so the recruitment message was addressed to both parents and teens. This message directed those interested in participating to a REDCap survey, which screened for additional inclusion criteria, including vaping status. Teens eligible for participation, according to their survey responses, were asked for their contact information, preferred contact time, and methods for scheduling a consent call with both parent and child.

*Selection criteria*

To ensure the sample represents diverse demographic groups, all adolescents aged 12–17 years were eligible for this study. We did not exclude participants based on national origin, sexual orientation, or socioeconomic status.

Adolescents who did not consent to participate in the study and those who did not speak English were excluded. Adolescents with asthma were recruited through EHR using ICD-10 codes J45 (see Supplementary file Material 3). A snowball sampling method was conducted to invite siblings of asthmatic adolescents to participate in the study in the ‘no asthma’ group. Children recruited by the ICD-10 code but self-reported having no asthma were also classified into the ‘no asthma’ group.

### Step 3: Study design and program assessment

We applied a prospective, interventional, and single-arm design to evaluate the effectiveness of the MediaSense program on the changes in media literacy, vaping expectancy, harm perception, and vaping susceptibility. The pilot study was approved by the Institutional Review Board at the University of Nebraska Medical Center.

All participants were informed about confidentiality and the voluntary nature of the study. As part of the informed consent process, parents or legal guardians of the youth participants provided consent, acknowledging their understanding of the study’s purpose, procedures, potential risks, and benefits. Additionally, the youth participants aged 12–17 years provided their assent, indicating their willingness to participate in the study after receiving age-appropriate information about the study.

Data were collected before and after the MediaSense intervention using REDCap with a URL link emailed to youth participants. To avoid bias from social desirability, participants could self-administer the e-module and complete the online survey anonymously without parental oversight. Participants received a $15 reward card for the pre-survey, $10 for each of the first two lesson surveys, and $15 for the final post-survey, totaling $50 as compensation for their time completing the surveys.

*Demographics assessed at the pre-intervention*

Demographic variables were assessed at baseline, including sex (female, male), grade level (middle school, high school), and race/ethnicity (non-Hispanic White, non-Hispanic Black, Hispanic, or Other). In addition, participants self-reported e-cigarette use status (ever vs never), peer vaping status (none, some, most, or all), and history of asthma (yes, no) in the survey. To ensure a sufficient sample size, this study combined non-Hispanic Black, Hispanic, and Other into a non-White category in the analysis.

*Measures assessed at both pre- and post-intervention*

Vaping media literacy

We assessed vaping media literacy using a validated scale covering three subdomains: Authors and Audience (vAA), Messages and Meanings (vMM), and Representation and Reality (vRR), each with two items on a four-point Likert scale (strongly agree, agree, disagree, strongly disagree). The vaping media literacy scale consists of six items. vAA1 (Target): E-cigarette companies create their ads to target teens. vAA2 (Appeal): Certain e-cigarette brands are specifically designed to appeal to kids and teens. vMM1 (Desire): E-cigarette ads associate vaping with natural human desires, such as love, good looks, and power. vMM2 (Influence): People are influenced by advertising, whether they realize it or not. vRR1 (Reframe): Most e-cigarette commercials and ads that depict people vaping make vaping appear more attractive than it actually is. vRR2 (Mislead): E-cigarette ads often omit important information, such as health risks. We converted responses into a binary variable: 1 (Strongly agree) and 0 (Agree, Disagree, Strongly disagree). We adopted this binary classification approach to distinguish strong endorsement of vaping media literacy concepts from all other levels of agreement or disagreement, which had been validated to be predictive of vaping prevention outcomes^5^. Scores for each subscale (vAA, vMM, vRR) were summed, ranging from 0 to 2. The composite vaping media literacy score, ranging from 0 to 6, was the sum of the subscale scores^5^.

**Figure 1:**
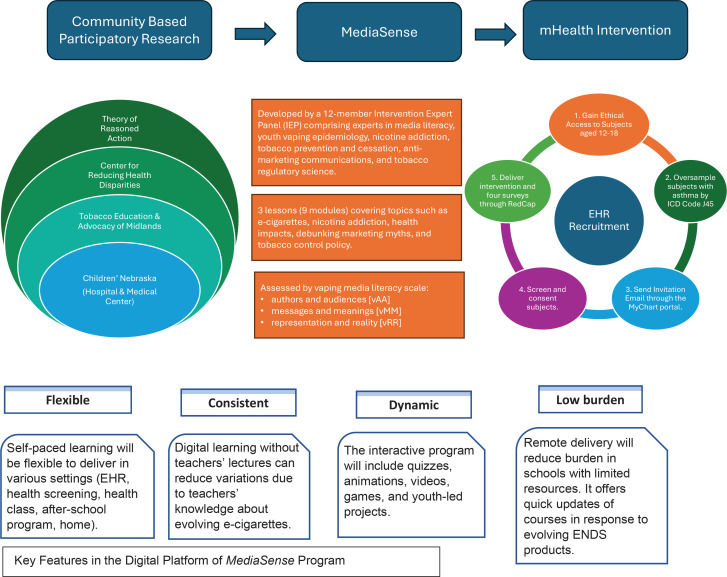
Logic model for media literacy e-cigarette program via e-learning and EHR recruitment

Vaping expectancy

Vaping expectancy was adapted from existing instruments that measure vaping outcome expectancies^17^ and is measured by a composite score from six items, including: ‘If I vape, I will enjoy it’; ‘If I vape, it will help me to deal with my problems and stress’; ‘If I vape, it will make me look cool among friends’; ‘If I vape, it will make me alert and energized’; ‘If I vape, it will increase my athletic performance’; and ‘If I vape, it will help me concentrate at school’. Each statement is rated on a 4-point Likert scale from 1 (strongly disagree) to 4 (strongly agree), and the mean score reflects the strength of positive vaping expectancies.

Harm perception of e-cigarette use

Participants were asked: ‘How much harm do you think e-cigarettes cause when they are used only some days but not every day?’, the response ‘a lot of harm’ was coded as 1 (harmful perception), while the other three options (i.e. some harm, little harm, no harm) were coded as 0 (not harmful perception)^5,18^.

Susceptibility to e-cigarette use

Susceptibility to e-cigarette use consists of two validated items: 1) ‘Do you think you will use an e-cigarette in the next year?’; and 2) ‘If one of your best friends were to offer you an e-cigarette, would you use it?’. Responses were categorized into a dichotomous variable: ‘Not susceptible to e-cigarette use’ (coded as 0) for those answering ‘Definitely not’ to both questions and ‘Susceptible to e-cigarette use’ (coded as 1) for all other responses^5,19^.

### Measures assessed at the post-intervention

Usability m easures of MediaSence e-modules

After taking the lessons, participants were assessed with a total of twenty-two usability measures regarding the MediaSense e-modules, with the 5-point Likert scale ranging from strongly disagree to strongly agree (Table 2). These measures were adapted from our prior studies and literature, including validated scales such as the System Usability Scale^20,21^.

**Table 1:** Changes in media literacy and perception before and after the MediaSense intervention

*Primary and secondary outcomes*	*Pre intervention Mean (SD)*	*Post intervention Mean (SD)*	*Fold change^b^ %*	*Adjusted p^c^*
*Vaping media literacy^a,d^*				
**Overall 6-item** (primary)				
(vAA+vMM+vRR, 0–6)	2.9 (2.1)	4.5 (2.1)	155	<0.0001
**vAA: Authors and Audiences**				
vAA subscale (0–2)	0.8 (0.9)	1.5 (0.8)	188	0.0007
vAA1: Target (0–1)	0.4 (0.5)	0.8 (0.4)	200	0.008
vAA2: Appeal (0–1)	0.4 (0.5)	0.7 (0.5)	175	0.03
**vMM: Messages and Meanings**				
vMM subscale (0–2)	1 (0.8)	1.4 (0.8)	140	0.03
vMM1: Desire (0–1)	0.4 (0.5)	0.8 (0.4)	200	0.007
vMM2: Influence (0–1)	0.6 (0.5)	0.6 (0.5)	100	0.69
**vRR: Representation and Reality**				
vRR subscale (0–2)	1.2 (0.8)	1.6 (0.7)	133	0.06
vRR1: Reframe (0–1)	0.5 (0.5)	0.8 (0.4)	160	0.05
vRR2: Mislead (0–1)	0.6 (0.5)	0.8 (0.4)	133	0.43
*Vaping perception*				
Vaping expectancy^e^ (primary)	3.6 (0.5)	1.2 (0.4)	33	<0.0001
Susceptibility to vaping (%)^f^	18.6	9.1	49	0.4
Perceived vaping harmfulness (%)^f^	40.3	86.0	213	<0.0001

a The vaping media literacy is a 6-item scale. vAA1 (Target): E-cigarette companies create their ads to target teens. vAA2 (Appeal): Certain e-cigarette brands are specially designed to appeal to kids/teens. vMM1 (Desire): E-cigarette ads link vaping to natural things that humans want, like love, good looks, and power. vMM2 (Influence): People are influenced by advertising whether they realize it or not. vRR1 (Reframe): Most e-cigarette commercials and ads that show people vaping make vaping look more attractive than it really is. vRR2 (Mislead): E-cigarette ads usually leave out a lot of important information (such as health risks).

b Fold change = post-intervention score/pre-intervention score.

c The dependent variables are listed in the first column, and the intervention served as the predictor variable. P-values for intervention were adjusted by sex, grade, race/ethnicity, and asthma status.

d Count dependent variables were analyzed using Poisson regression.

e Continuous dependent variable was analyzed using linear regression.

f Binary dependent variables were analyzed using logistic regression.

**Table 2:** Usability and satisfaction assessment with MediaSense through interactive e-learning

*E-Learning Usability Evaluation Questionnaire items*	*Usability assessment score^a^*				*Factor analysis^c^*		
	*Overall Mean (SD)*	*No Asthma Mean (SD)*	*Asthma Mean (SD)*	*p^b^*	*Factor 1*	*Factor 2*	*Communality*
I would like to use e-modules like this lesson to learn other things in the future	3.9 (0.9)	3.6 (0.9)	4.2 (0.8)	0.007	-0.09	**0.60**	0.30
It was easy to use this e-module	4.4 (0.6)	4.2 (0.5)	4.6 (0.6)	0.006	0.30	**0.58**	0.64
I needed someone to help me understand how to use this e-module.	1.6 (0.9)	1.6 (0.7)	1.5 (1.0)	1	-0.15	-0.13	0.06
It was easy to use the menu and buttons in this e-module	4.3 (0.7)	4 (0.8)	4.5 (0.7)	0.03	0.14	**0.61**	0.49
I got lost or may have accidentally skipped content when trying to complete this lesson	1.7 (0.9)	1.8 (0.9)	1.7 (0.9)	0.65	-0.03	-0.25	0.07
Most people my age would quickly figure out how to use this e-module.	4.4 (0.9)	4 (1.0)	4.6 (0.7)	0.01	-0.14	**0.56**	0.23
I liked how this e-module was designed	4.1 (0.9)	3.7 (1.0)	4.4 (0.7)	0.004	0.16	**0.77**	0.77
I felt confident using this e-module	4.3 (0.7)	4 (0.8)	4.5 (0.7)	0.01	0.28	**0.67**	0.76
The language used in the lessons is easy to understand	4.6 (0.6)	4.5 (0.5)	4.7 (0.6)	0.15	0.37	0.19	0.26
The design and layout of the lessons made it easy to read the material	4.4 (0.8)	4.1 (0.9)	4.6 (0.7)	0.01	**0.59**	0.30	0.66
The font and text are clear and easy to read	4.6 (0.7)	4.4 (0.7)	4.6 (0.6)	0.15	**0.89**	-0.13	0.67
Information is easy to follow and wellorganized	4.5 (0.6)	4.4 (0.5)	4.6 (0.6)	0.09	**0.84**	0.02	0.74
It is easy to move through the content of the lessons	4.5 (0.7)	4.4 (0.7)	4.6 (0.6)	0.13	**0.85**	-0.01	0.72
I completed the lessons with little or no help from others	4.7 (0.7)	4.5 (0.8)	4.8 (0.6)	0.16	**0.65**	0.09	0.50
The pace and speed of the lessons are just about right	4.4 (0.8)	4 (0.9)	4.6 (0.7)	0.01	**0.57**	0.30	0.62
The time to complete one lesson is appropriate (neither too long nor too short)	4.3 (0.8)	4.1 (0.9)	4.5 (0.8)	0.06	0.45	0.31	0.47
I liked the images, videos, and animations in the lesson	4.2 (0.8)	3.8 (1.0)	4.5 (0.7)	0.005	**0.83**	0.00	0.69
The videos in the lessons helped me understand the concepts	4.4 (0.8)	4.1 (0.8)	4.5 (0.8)	0.07	**0.99**	-0.11	0.86
The animations in the lessons helped me understand the concepts	4.4 (0.7)	4.1 (0.7)	4.6 (0.7)	0.01	**0.89**	-0.03	0.76
The quizzes in the lessons helped me understand the concepts	4.3 (0.9)	4.1 (0.7)	4.4 (0.9)	0.23	**0.78**	0.07	0.69
I understand the concepts of each lesson clearly	4.6 (0.7)	4.2 (0.7)	4.8 (0.6)	0.003	**0.77**	0.09	0.69
These lessons increased my knowledge about vaping	4.4 (0.8)	4.1 (0.9)	4.6 (0.7)	0.02	**0.74**	0.05	0.60

a The usability of MediaSense iterative e-learning was assessed by a 5-point Likert scale: 1-strongly disagree, 2-disagree, 3-neutral, 4-agree, 5-strongly agree.

b General linear model was performed to compare the learning measures by asthma status, adjusted by sex, grade, and race/ethnicity.

c Rotated factor pattern (standardized regression coefficients) is reported for the factor analysis of e-learning usability measures. A magnitude of ≥0.5 (bold) indicates a salient variable-factor relationship. Factor 1 features clear and convincing content. Factor 2 summarizes the user-friendly, intuitive design. Communality ranges from 0 to 1, with higher scores indicating that a greater proportion of the variable’s variance is explained by the factors.

Vaping refusal and knowledge

**Figure 2:**
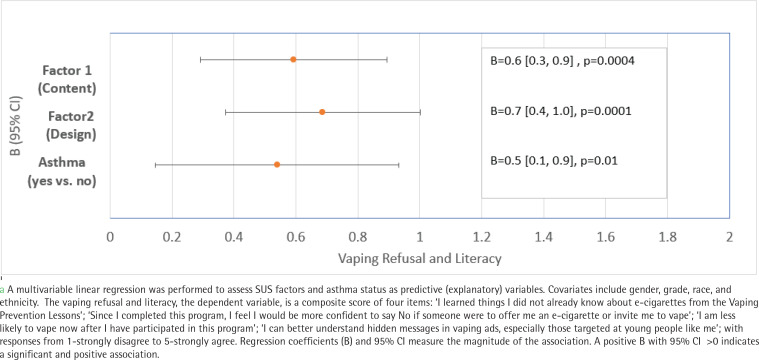
Protective factors associated with improved vaping prevention outcomes after MediaSense intervention^a^

The vaping refusal and knowledge is a composite score of four questions (e.g. ‘I am less likely to vape now after I have participated in this program’). See Figure 2 footnote with responses from 1-strongly disagree to 5-strongly agree.

### Statistical analysis

Descriptive analyses, including mean, standard deviation (SD), and percentage, were reported for the sample characteristics and intervention measures. Generalized estimating equation models were used to examine changes in vaping media literacy (the primary intervention outcome), vaping expectancy, harm perception related to vaping, and susceptibility to e-cigarette use before and after the intervention while accounting for repeated measures of individuals over time. For the 6-item vaping media literacy scale, changes in scores for individual items, subscales, and the overall composite score were analyzed. Binary outcome variables were analyzed using a binomial distribution with a logit link function and continuous outcomes were analyzed using a linear regression, while Likert scale-based outcomes were treated as count variables and analyzed using a Poisson distribution with a log link function.

Factor analysis of 22 usability measures of MediaSence e-modules was performed using an oblique rotation method (rotate = promax), allowing for correlated factors^22^. This comprehensive factor analysis aimed to identify the underlying structure of these variables and reduce the data dimensionality while preserving as much information as possible. The number of factors was determined using a scree plot. Composite scores for each factor were calculated by summing the usability measures with loadings >0.5 indicating a salient relationship between the variable and the factor. Finally, a regression analysis was performed to assess associations of latent factors and vaping refusal and knowledge. All analyses were conducted using SAS 9.4 (SAS, Cary, NC, USA) with statistical significance at p<0.05. All multivariable regression analyses were adjusted by sex, grade, race/ethnicity, and asthma status.

## RESULTS

### Sample characteristics

In total, 67 adolescents aged 12–17 years participated in the pilot study [mean age 14.3 years (SD=1.5), median age 14.0 years]. We intentionally oversampled participants with asthma (n=40; 59.7%) (Supplementary file Material 2). The sample included a balanced number of middle and high school students. At baseline, most participants had never used e-cigarettes or other tobacco products, although 26.9% reported that their peers vaped.

### Effectiveness of MediaSense intervention

As shown in Table 1, the mean scores of vaping media literacy, both collectively and individually, increased significantly from pre- to post-intervention. There was a 148% fold change (FC) in the overall mean score (2.9 vs 4.5; p<0.0001). Similar magnitudes of increases were observed in the subscales: vAA (FC=188%, p=0.0007), vMM (FC=140%, p=0.03), and vRR (FC=133%, p=0.06).

For vaping prevention outcomes, positive vaping expectancy decreased significantly from 3.6 to 1.2 (p<0.0001), and the perception of vaping as harmful increased significantly from 40.3% to 86.0% (p<0.0001) before and after the intervention. The susceptibility to vaping also fell from 18.6% to 9.1% though it was not statistically significant (p=0.4).

### Usability of interactive e-learning

Participants rated the MediaSense intervention highly on general usability, technical assistance, design and layout, content clarity, navigation, flow, multimedia, interactivity, and learning outcomes. On a 5-point Likert scale, the highest rated items included ‘completed the lessons independently’ (mean score 4.7), ‘clearly understood the concepts’ (4.6), and ‘easy to use’ (4.4).

Participants with asthma rated the course higher in several measures related to MediaSense intervention compared to those without asthma, including ‘use e-modules like this lesson to learn other things in the future’ (4.2 vs 3.6; p=0.007), ‘liked how this e-module was designed’ (4.4 vs 3.7; p=0.004), and felt ‘these lessons increased my knowledge about vaping’ (4.6 vs 4.1; p=0.02).

### Factor analysis

Twenty-two usability items were analyzed to assess the usability of interactive e-learning modules. Two distinct latent factors were identified: 1) Motivating and engaging content (12 items, e.g. ‘Information is easy to follow and well-organized’, ‘The videos in the lessons helped me understand the concepts’, and ‘The animation in the lessons helped me understand the concepts’); 2) User-friendly module design (6 items, e.g. ‘It was easy to use the menu and buttons in this e-module’, ‘I liked how this e-module was designed’, ‘I felt confident using this e-module’).

Regression analysis in Figure 2 shows that both latent factor 1 (motivating and engaging content) and latent factor 2 (user-friendly module design) were significantly associated with a higher mean score of vaping refusal and literacy (B=0.6; 95% CI: 0.3–0.9, p=0.0004; and B=0.7; 95% CI: 0.4–1.0, p=0.0001; respectively). Participants with asthma also reported a higher mean score of vaping refusal and literacy compared to those without asthma (B=0.5; 95% CI: 0.1–0.9, p=0.01).

## DISCUSSION

This pilot study demonstrates the feasibility of a vaping prevention program that incorporates vaping marketing strategies and tactics as core components and recruitments from the EHR system. The findings highlight the program’s positive impact on media literacy, harm perception, and vaping expectancy. The digitally delivered MediaSense intervention resulted in increased overall media literacy scores and improved scores in each subdomain (vAA, vMM, and vRR).

The pediatric primary care setting offers excellent opportunities to screen for youth vaping, and healthcare providers play a crucial role in advising youth not to use tobacco products. Although both the US Preventive Services Task Force^23^ and the American Academy of Pediatrics^24^ recommend that healthcare professionals provide clinical tobacco screening and prevention interventions to youth, less than one-third of US middle and high school students reported e-cigarette-specific screening by their healthcare providers in the past 12 months^25^, representing a missed opportunity for youth’s vaping prevention. The low prevalence of health professional screening for e-cigarette use may relate to limited time during clinical visits, reduced priority on tobacco avoidance, limited knowledge about e-cigarettes, and perceptions that cessation treatments for adolescents show limited efficacy^25,26^.

This study successfully leveraged the EHR system to recruit adolescent patients, including those with asthma identified through ICD-10 codes, demonstrating its feasibility for vaping interventions. The EHR system offers several advantages: 1) High reach, as its use has expanded in primary care and tobacco control research; 2) Enhanced sampling design, allowing researchers to identify racial minorities and underserved populations; 3) Cost efficiency, reducing recruitment time and expenses; 4) High acceptability and retention, as healthcare providers are trusted sources; and 5) Scalability, enabling integration into pediatric clinical settings. In summary, the EHR system provides a cost-effective and replicable approach to engaging vulnerable adolescents in vaping interventions^27,28^.

Additionally, interactive e-learning offers several advantages for vaping prevention among adolescents. It enhances engagement through multimedia elements, quizzes, and simulations, making learning more appealing and effective than traditional methods. The convenience of accessing content anytime and anywhere allows adolescents to learn at their own pace, thus providing a scalable solution without the constraints of physical classrooms. This study further analyzed twenty-two usability items and evaluated which factors were most relevant to effective e-learning modules. Two factors emerged through the factor analysis including motivating and engaging content, and user-friendly module design, and both factors were associated with higher vaping refusal and knowledge through the intervention. Recently adolescent vaping programs that are built on a mobile app, social media sites (e.g. Instagram, and peer-to-peer text messaging, e.g. Quit the Hit, Vaper to Vaper) have recently emerged^29,30^. However, the adoption of the vaping digital intervention program is still relatively low^31^. Findings from our study provided insightful information to inform the development of successful digital vaping interventions that can be delivered through medical record systems. By understanding key factors related to e-learning engagement, these insights contribute to creating targeted content and strategies that enhance the success and impact of digital approaches in addressing vaping behavior.

Adolescents with chronic medical conditions might be more vulnerable to vaping, which could further increase the risk of other tobacco or substance use. Chronic conditions, such as asthma, COPD, and depression, are common among children, and the prevalence of chronic medical conditions nearly quadrupled from 1.8% in 1960 to 7.0% in 2005^32^. In recent years, it is estimated that 10–30% of youth have a chronic health condition^33^. Recruitment through EHR offers a unique opportunity to reach adolescents with chronic medical conditions. Our study is one of the first to show that youth with asthma were highly engaged with digitally delivered MediaSense, which seeks to address the extensive marketing and promotional efforts by EC manufacturers^34^. Exposure to EC marketing messages has been associated with increased intentions to use and willingness to pay more for these products^3,35^. After MediaSense intervention, youth with asthma can have a tool to debunk marketing strategies, which could enhance vaping expectancy, especially considering the influential role of social media on their behavior. Therefore, incorporating media literacy into vaping prevention and control programs holds promise, as it equips adolescents with essential skills to comprehend, analyze, and counter media messages.

### Limitations

Several limitations should be acknowledged in this study. Firstly, recruitment through EHR is the first step to reach a large number of adolescents for vaping prevention. Future research should consider implementing the MediaSense program alongside youth e-cigarette screening in clinical settings. Additionally, a cluster randomized control trial^36^ across multiple centers and a broader geographical region is needed to validate the pilot test findings and assess the program’s robustness. Secondly, self-reported measures may be subject to social-desirability bias. However, we mitigated this by conducting surveys confidentially and anonymously. Surveys were directly emailed to students, allowing them to complete evaluations privately, without influence from parents or teachers. Additionally, clear instructions emphasized the importance of honesty to improve accuracy.

## CONCLUSIONS

Our preliminary work (Phase I) with the MediaSense program has shown promising outcomes in reducing vaping susceptibility by improving adolescents’ vaping media literacy in school settings^16^. This study (Phase II) further explores reaching adolescents through EHR, using ICD-10 codes to identify patients with asthma and potentially other chronic diseases in the future. The interactive e-learning allows youth to access e-modules independently, offering a cost-effective and comprehensive approach to anti-marketing education. This method helps adolescents understand tobacco marketing tactics and address emerging issues, providing valuable evidence for reducing vaping behavior among teenagers.

## Supplementary Material



## Data Availability

The data supporting this research are available from the authors on reasonable request.
