# Supplementary material for: Developing a media literacy-based e-cigarette education program via medical record systems
Source: Tob Prev Cessat. 2025 Apr 4;11:10.18332/tpc/201477. doi: 10.18332/tpc/201477 (PMC11956842; doi:10.18332/tpc/201477)
Supplement: Supplementary file 1 [file TPC-11-22-s1.pdf]

Appendix 1. MediaSense Development

Media Education for Sensible Evaluation and Nurturing Substance-free Experiences (MediaSense) is an anti-vaping media literacy program designed to prevent vaping among adolescents and young adults.[16] Following CDC and FDA guidelines, MediaSense was developed using a community-based participatory approach in collaboration with two Nebraska tobacco coalitions: Tobacco Education & Advocacy of Midlands (TEAM) and the Center for Reducing Health Disparities (CRHD) at the University of Nebraska Medical Center (UNMC). These long-term collaborations ensure the program's sustainability and effective community outreach.

Theoretical Framework

The program is based on the Theory of Reasoned Action (TRA), which links intention and behavior influenced by marketing and media.[14,15] Initially used for smoking-related media literacy, this framework was adapted for e-cigarettes, considering differences in product makeup and usage patterns. TRA posits that a person's behavior is driven by their intention, influenced by attitudes and perceived social norms. Adolescents exposed to e-cigarette marketing may see vaping as socially acceptable, fostering pro-vaping attitudes and increasing their likelihood of starting. Additionally, marketing exposure may reduce perceived harm, thus increasing the vaping susceptibility.[36]

Phase I Development

In Phase I, three major tasks were completed for program development. First, needs assessment and stakeholder engagement were conducted during a one-day retreat with over 50 superintendents, principals, teachers, and social workers. Focus groups were held to gather information on intervention scope, sequence, delivery mode, and implementation barriers. Second, a 6-item vaping media literacy (vML) scale was adapted from validated smoking (n=11 items) and general media literacy (n=11 items) scales, focusing on marketing influences. The reliability of the vML and its three subscales (authors and audiences [vAA], messages and meanings [vMM], and representation and reality [vRR]) was refined after a thorough literature review to assess marketing differences between cigarettes and e-cigarettes. Third, the program's effectiveness was tested in schools, showing significant improvements in vaping media literacy across all three

domains. Causal mediation analysis revealed that the intervention reduced vaping susceptibility by enhancing media literacy and harm perception.

Appendix 2. Sample Characteristics

Characteristics	n	%
Overall	67	100
Sex		
Male	38	57.6
Female	28	42.4
Grade		
Middle School	32	47.8
High School	35	52.2
Race / Ethnicity		
NH-white	60	89.6
Non-White	7	10.4
E-cigarette Ever Use[a]		
No	59	88.1
Yes	8	11.9
Other Tobacco Ever Use[b]		
No	59	88.1
Yes	8	11.9
Peer E-cigarette Use[c]		
None	41	63.1
Some/Most/All	24	26.9
Asthma Status		
No	27	40.3
Yes	40	59.7

[a] E-cigarette ever use was assessed by the question “Have you ever used an e-cigarette such as JUUL, Puff Bar, NJOY, Blu, VUSE, MarkTen, Logic, Vapin Plus, eGo, or Halo, even once or twice?”

[b] Other tobacco ever use was assessed by two questions: “Have you ever smoked a cigarette, even one or two puffs?” and “Have you ever used other tobacco products, such as cigars (cigars, little cigars, and cigarillos), smokeless tobacco (chewing tobacco, snuff, dip, snus, and dissolvable tobacco), hookahs, pipe tobacco, bidis, even one or two puffs?”

[c] Peer e-cigarette use was assessed by the question, “Do any of your closest friends use e-cigarettes?”

Appendix 3. Study Protocol

ICD 10 codes of asthma

- J45.2 Mild intermittent asthma.
- J45.20 Mild intermittent asthma, uncomplicated. ...
- J45.3 Mild persistent asthma.
- J45.30 Mild persistent asthma, uncomplicated. ...
- J45.4 Moderate persistent asthma.
- J45.40 Moderate persistent asthma, uncomplicated. ...
- J45.5 Severe persistent asthma. ...
- J45.9 Other and unspecified asthma. ...
- J45.99 Other asthma.

Selection Criteria

- Adolescents aged 12–17 years.
- Adolescents from diverse demographic groups, including all national origins, sexual orientations, and socioeconomic statuses.
- Adolescents with asthma, identified through EHR using ICD-10 code J45.

- Siblings of asthmatic adolescents, recruited into the "no asthma" group via snowball sampling.

Exclusion Criteria

- Adolescents who did not consent to participate in the study.
- Adolescents who do not speak English.

Step 1:

The research team will have ethical access to patients. A recruitment/screening email will be sent to patients aged 12-17 with an ICD Code of J45 through the "MyChart" patient portal at the Children's Nebraska. If an interested patient contacts the research team because of the recruitment flyer or other mode outside of the recruitment email, the interested patient will be sent the recruitment/screening email to keep the consenting process consistent throughout the study. The recruitment/screening email will include a link to a REDCap survey that participants will choose if they are interested in pursuing the study. Based on expressed interest in participating through the survey interest form, we will recruit 50-100 subjects to participate in the study.

Step 2:

If a patient selects that they are interested in participating, they will follow the link in the recruitment/screening email to a REDCap. If interested, participants will then be asked to give permission so that a member of the research team can contact the patient and their parent/legal guardian to give their consent to participate in the study. Patients will also be asked which method of remote consent (phone or Zoom) they would prefer and to also provide several times and days that the patient and their parent/legal guardian would both be available to speak to give their consent.

Step 3:

Based on the preferred method of communication, a member of the research team will contact the patient and their parent/legal guardian to obtain verbal consent. The research team member will use a pre-written script for consent (speaking with parent/legal guardian) and assent (speaking with the patient) to describe the study, why this patient was chosen to participate (inclusion criteria),

explain the incentive procedure, remind participants that they can stop at any time, and answer questions.

Step 4:

After obtaining consent and assent, the research team will:

1) Obtain protected health information (PHI) and assign a unique research ID number to be used for data collection.
2) Send a REDCap Pre-survey to subjects. Upon completion of the pre-survey, participants will earn a $15 reward card.

Step 5:

3) A day after completion of the baseline survey, participants will be sent the first lesson video to watch and will complete a short survey to ensure the completion of the video. Upon completion of the short post-lesson survey, participants will earn a $10 reward card.
4) A day after completion of the lesson 1 survey, participants will be sent the second lesson video to watch and will complete a short survey to ensure the completion of the video. Upon completion of the short post-lesson survey, participants will earn a $10 reward card.
5) A day after completion of the lesson 2 survey, participants will be sent the third lesson video to watch and will complete a post-survey. Upon completion of the post-survey, participants will earn a $15 reward card.
6) We will send reminder emails to participants who do not complete their lesson within a week after being sent the lesson. If we see that the participant has not completed the lesson after the reminder email has been sent, then we may call the participant to remind them to complete the lesson.

Study Overview:

- Participants will be asked to complete an online pre-survey that will assess participant knowledge, attitudes, and use of e-cigarettes. In addition to this, with prior consent and using patient charts, we will collect the following measurements from EPIC for the following demographic and asthma data: Name, Date of Birth, Age, Sex, Race/Ethnicity,

Address, Email, Primary Language, Telephone Number, CASI, HealthSence (Confidence Score), tobacco use status, Spirometry: FVC%, FEV1%, FEV1/FVC, FeNO.

- Participants will then be asked to complete and watch a 3-part educational lesson explaining what an e-cigarette is, the harms of e-cigarettes, and how e-cigarettes are marketed to young adults.
- Once the three lessons have been completed, participants will complete an online post-survey to assess participant knowledge, attitudes, and use of e-cigarettes.

© 2025 Dai H.D. et al.
